# HMGB1 release by H_2_O_2_-induced hepatocytes is regulated through calcium overload and 58-F interference

**DOI:** 10.1038/cddiscovery.2017.8

**Published:** 2017-04-10

**Authors:** Pei Zhao, Tingjie Ye, Xiaofeng Yan, Xudong Hu, Ping Liu, Xiaoling Wang

**Affiliations:** 1 The Public Experiment Platform, School of Basic Medicine, Shanghai University of Traditional Chinese Medicine, Shanghai 201203, China; 2 Department of Biology, School of Basic Medicine, Shanghai University of Traditional Chinese Medicine, Shanghai 201203, China; 3 Key Laboratory of Liver and Kidney Diseases (Ministry of Education), Institute of Liver Diseases, Shuguang Hospital, Shanghai University of Traditional Chinese Medicine, Shanghai 201203, China; 4 E-institute of Shanghai Municipal Education Commission, Shanghai 201203, China

## Abstract

HMGB1 is passively released by injured or dying cells and aggravates inflammatory processes. The release of HMGB1 and calcium overload have each been reported to be important mediators of H_2_O_2_-induced injury. However, a potential connection between these two processes remains to be elucidated. In the present study, we employed H_2_O_2_-induced hepatocytes to investigate how calcium overload takes place during cellular injury and how the extracellular release of HMGB1 is regulated by this overload. In addition, we investigated the use of 58-F, a flavanone extracted from *Ophiopogon japonicus,* as a potential therapeutic drug. We show that the PLC*γ*1–IP_3_R–SOC signalling pathway participates in the H_2_O_2_-induced disturbance of calcium homoeostasis and leads to calcium overload in hepatocytes. After a rise in intracellular calcium, two calcium-dependent enzymes, PKC*α* and CaMKIV, are activated and translocated from the cytoplasm to the nucleus to modify HMGB1 phosphorylation. In turn, this promotes HMGB1 translocation from the nucleus to the cytoplasm and subsequent extracellular release. 58-F effectively rescued the hepatocytes by suppressing the PLC*γ*1–IP_3_R–SOC signalling pathway and decreasing the calcium concentration in cells, thus reducing HMGB1 release.

## Introduction

Calcium is a universal second messenger involved in a remarkably wide range of cellular processes.^1^ Disordered cytosolic calcium signalling can lead to severe damage or result in cell death.^2,3^ In non-excitable cells, Ca^2+^ signals are generated by the phospholipase C (PLC)-mediated hydrolysis of phosphatidylinositol bisphosphate (PIP_2_) to yield 1,4,5- trisphosphate (IP_3_), leading to the subsequent activation of the inositol trisphosphate receptor (IP_3_R). This mediates the release of Ca^2+^ from the endoplasmic reticulum (ER),^4^ followed by transmembrane Ca^2+^ entry through the opening of store-operated calcium (SOC) channels.^5^ SOC channels are the predominant mechanism of calcium entry in both excitable and non-excitable cells and are activated by the depletion of internal calcium stores, for example, from the ER. Upon opening, SOC channels promote calcium entry through the plasma membrane (PM), a major mechanism for Ca^2+^ influx.^6,7^ So far, two major molecular components of the SOC channel signalling pathway have been identified: stromal interaction molecule 1 (STIM1) and Orai1.^8,9^ STIM1 serves as a calcium sensor that can directly bridge the ER to PM at specialized junctions, aggregating into puncta in response to calcium store depletion and triggering the activation of SOC channels located in the PM.^10^ Orai1 channels comprise six monomers and are localized diffusely in the PM of resting cells. They are recruited into puncta by STIM1 through a direct interaction, opening SOC channels to mediate store-operated calcium entry (SOCE) to ensure the optimal refilling of the ER.^11,12^ SOCE plays a major role in Ca^2+^ influx in non-excitable cells, including hepatocytes.^13^ Patch-clamp experiments in liver cells showed that only one type of SOC channel, a highly Ca^2+^-selective channel, could be detected.^14,15^ Therefore, SOC channels are a research hotspot in the physiology and pathology of liver disease.

Hydrogen peroxide (H_2_O_2_) is a key mediator underlying cellular oxidative stress and is involved in a wide variety of pathological processes. It can cause intracellular Ca^2+^ overload in various cell types due to oxidative stress.^16–18^ Therefore, any therapeutic approach that can prevent H_2_O_2_-induced intracellular Ca^2+^ overload and improve intracellular Ca^2+^ regulation would be beneficial for cells. On the other hand, PLC*γ*1, the first protein in the PLC*γ*1–IP_3_R–SOC Ca^2+^ signalling pathway, is upregulated as a result of H_2_O_2_-induced oxidative stress. PLC isozymes are subdivided into three types (*β*, *γ*, *δ*), and the *γ* type includes two isoforms (PLC*γ*1 and PLC*γ*2). While PLC*γ*1 shows a ubiquitous expression pattern, PLC*γ*2 is mainly expressed in B-cells.^19^ Moreover, it is well established that PLC*γ*1 undergoes direct phosphorylation on tyrosine residues in response to H_2_O_2_ treatment.^20,21^ However, to the best of our knowledge, whether PLC*γ* is involved in H_2_O_2_-induced Ca^2+^ release in hepatocytes remains unknown.

High mobility group box 1 (HMGB1) is a highly conserved 30 kDa DNA-binding protein. In response to injury, HMGB1 is passively released from stressed cells,^22^ and excessive extracellular HMGB1 adversely contributes to injury-elicited pathogenesis.^23^ In the same way, HMGB1 plays a key role in various forms of liver injury.^24^ The release of HMGB1 is controlled by two critical steps that regulate the flux of HMGB1 from the nucleus to the cytoplasm and subsequently from the cytoplasm to the extracellular compartment. The phosphorylation of HMGB1 at critical serine residues is essential for its translocation from the nucleus to cytoplasm.^25^ The activation of two calcium-mediated protein kinases, classical protein kinase C (PKC) and Ca^2+^/calmodulin-dependent protein kinase IV (CaMKIV), is required for HMGB1 phosphorylation.^26,27^ PKC has many isoenzymes, and among them, PKC*α* is involved in HMGB1 release.^26^ CaMKs are a family of proteins comprising CaMK I–IV. Activation typically requires Ca^2+^/calmodulin binding and can be augmented or sustained by phosphorylation. The identity of the specific CaMK family members involved in hypoxia-induced HMGB1 release is uncertain, but H_2_O_2_ treatment has been shown to activate CaMKIV in hepatocytes.^28^ In the present report, we employed these two calcium-dependent enzymes to determine whether they participate in HMGB1 phosphorylation associated with secretion.

Oxidative stress sensitizes hepatocytes to either calcium overload^29^ or HMGB1 release.^30^ Although HMGB1 and calcium have been separately reported to be important mediators of oxidative stress-induced injury, the potential relationship between them remains unknown. Here, we evaluate the hypothesis that SOCE activation causes calcium influx leading to calcium overload after H_2_O_2_ treatment, followed by the activation of PKC*α* and CaMKIV and HMGB1 release in hepatocytes. Our findings support this hypothesis, demonstrating that SOCE activation leads to a cytosolic calcium increase and HMGB1 release from cells after H_2_O_2_ treatment. In addition, the compound 5,8-dimethoxy-6-methyl-7-hydroxy-3-3(2-hydroxy-4-methoxybenzyl) chroman-4-one (58-F) is a flavanone extracted from *Ophiopogon japonicas,* which has been widely distributed and used clinically in mainland China,^29,31^ and we recently reported that 58-F protects against ROS-induced liver injury.^32^ In the present study, we explore the protective contribution of 58-F to H_2_O_2_-induced calcium homoeostasis and HMGB1 release.

## Results

### Release of HMGB1 following H_2_O_2_-induced hepatocyte injury/death is involved in calcium entry

**Figure 1:**
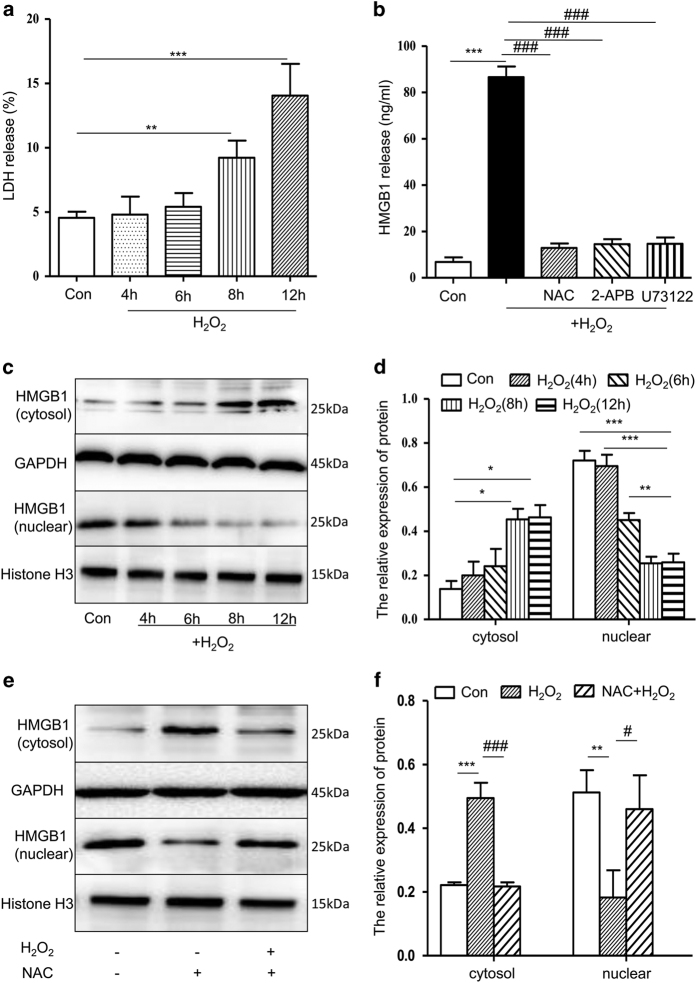
HMGB1 release induced by H_2_O_2_ is calcium dependent. (**a**) The rate of cell injury was measured using a Cytotoxicity LDH Assay Kit. (**b**) The cells were pre-treated with 100 *μ*g/ml NAC, 50 *μ*M 2-APB or 10 *μ*M U73122 for 16 h, then co-incubated with H_2_O_2_ for 8 h, and HMGB1 levels in media were measured by ELISA. (**c **and **d**) The cells were treated with 500 *μ*M H_2_O_2_, and a western blot was used to measure HMGB1 protein levels in the cytosolic/nuclear fractions at 4, 6, 8 and 12 h. (**e **and **f**) The cells were pre-treated with NAC for 16 h, then co-incubated with H_2_O_2_ for 8 h and HMGB1 levels in the cytosolic/nuclear were measured by western blot. Shown are representative results from one of three independent experiments. *P<0.05, **P<0.01, ***P<0.001, compared with the control; ^#^*P*<0.05, ^###^*P*<0.001, compared with the H_2_O_2_ treatment group.

Our previous studies found that H_2_O_2_ could induce apoptosis by disrupting cellular calcium homoeostasis.^33^ To confirm the effect of H_2_O_2_ on cell injury/death, we examined the release of lactate dehydrogenase (LDH) from cells to media as well as the levels of HMGB1 in the media, cytosol and nucleus. After incubation with 500 *μ*M H_2_O_2_ for varying times, the levels of LDH in the cellular media increased approximately 2.5 and 3-fold at 8 and 12 h, respectively (Figure 1a). HMGB1 is passively released to the extracellular space upon cellular injury/death by almost all cells that have a nucleus, and it acts as a signal to neighbouring cells of ongoing damage.^23^ Moreover, 500 *μ*M H_2_O_2_ led to an increase in HMGB1 release into the media (Figure 1b), and western blot analysis of cytosolic and nuclear lysates revealed that the amount of HMGB1 protein progressively increased in the cytosol and decreased in the nucleus after H_2_O_2_ treatment (Figures 1c and d). Pretreatment with 100 *μ*g/ml *N*-acetylcysteine (NAC), a common antioxidant, decreased the content of HMGB1 in the media (Figure 1b). However, HMGB1 levels in the cytosol and nucleus continued to trend the same way with or without the antioxidant (Figures 1e and f). Further, we tested the effects of calcium signalling pathway inhibitors on HMGB1 release. HMGB1 levels in the cellular media induced by H_2_O_2_ exposure were markedly reduced by pretreatment with 10 *μ*M U73122 (a PLC inhibitor) or 50 *μ*M 2-APB (an IP_3_R inhibitor) (Figure 1b). These findings indicate that cellular injury or death caused by H_2_O_2_ leads to the release of HMGB1 and that this process is regulated by a calcium signalling pathway.

### PLC*γ*1–IP_3_R–SOC participate in H_2_O_2_-induced calcium entry into cells

**Figure 2:**
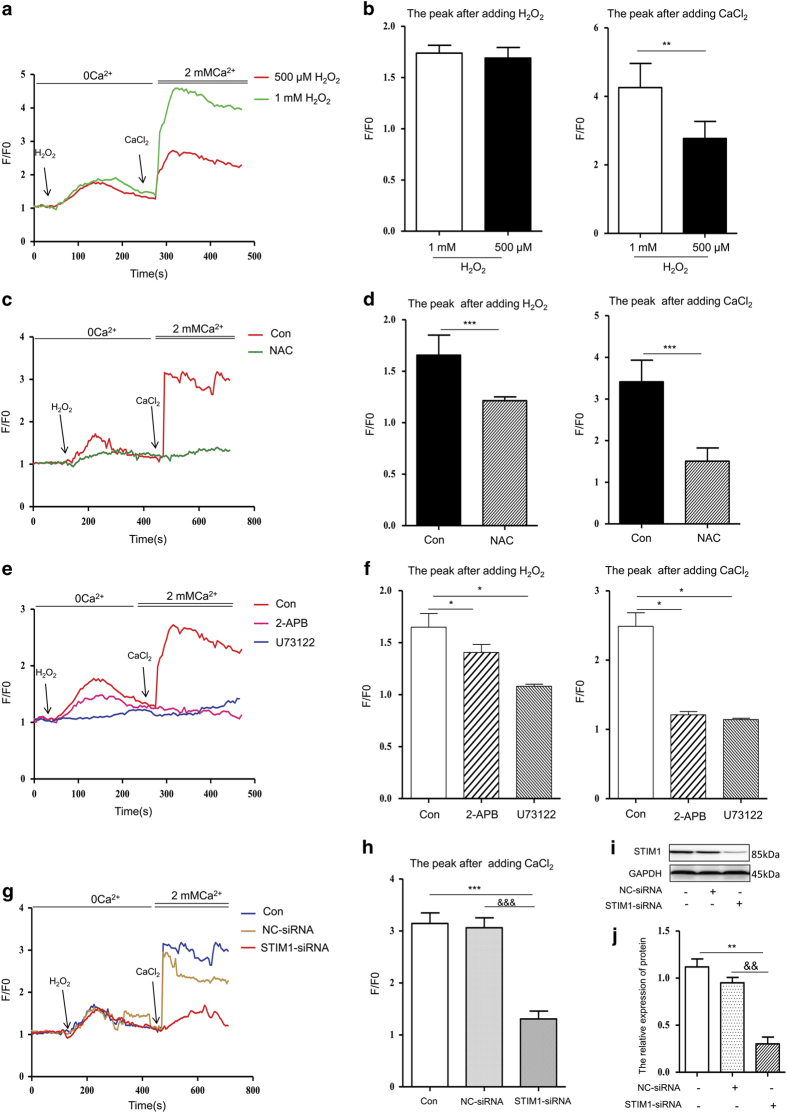
Changes in real-time Ca^2+^ fluorescence intensity induced by H_2_O_2_. (**a**) The cells were stimulated with 500 *μ*M and 1 mM H_2_O_2_ in Ca^2+^-free buffer, followed by the addition of 2 mM CaCl_2_ to the medium. (**c**) The cells were pre-treated with 100 *μ*g/ml NAC for 24 h, followed by stimulation with 500 *μ*M H_2_O_2_ and the subsequent addition of 2 mM CaCl_2_ to the medium. (**e**) The cells were pre-treated with 50 *μ*M 2-APB or 10 *μ*M U73122 for 24 h, followed by stimulation with 500 *μ*M H_2_O_2_ and the subsequent addition of 2 mM extracellular Ca^2+^. (**b**, **d** and **f**) The quantification of intracellular Ca^2+^ at the peak after adding H_2_O_2_ and CaCl_2_, respectively. (**g** and **h**) Changes in real-time Ca^2+^ fluorescence intensity of transfected cells induced by H_2_O_2_. Intracellular Ca^2+^ was quantified at the peak after adding CaCl_2_. (**i** and **j**) The expression of STIM1 in transfected cells detected by western blot. Shown are representative results from one of three independent experiments. *P<0.05, **P<0.01, ***P<0.001, compared with the control; ^&&^*P*<0.01, ^&&&^*P*<0.001, compared with the NC-siRNA.

To determine whether H_2_O_2_ affects calcium signalling in hepatocytes through SOC channels, we performed calcium imaging of hepatocytes stimulated with 500 *μ*M and 1 mM H_2_O_2_ in a calcium-free buffer (plus 1 mM EGTA). We observed an increase in the height of the left peaks representing Ca^2+^ transients in the cytoplasm, due to calcium release from intracellular store(s) for example, from the ER (Figure 2a). After replacing the extracellular medium with a 2 mM calcium chloride solution, an increase in the heights of the right peaks representing Ca^2+^ transients in the cytoplasm from media were observed (Figure 2a), indicating extracellular calcium influx through the PM due to the depletion of ER stores. Furthermore, NAC was used to assess whether the calcium influx was caused by oxidative stress, and we observed a significant reduction of H_2_O_2_-induced left and right peaks after NAC pretreatment (Figures 2c and d).

To investigate the possible involvement of PLC in H_2_O_2_-induced cytosolic calcium increases, cells were pre-treated with 10 *μ*M U73122 before H_2_O_2_ addition. The two peaks representing calcium entry into the cytoplasm were significantly reduced when cells were pre-treated with U73122 before H_2_O_2_ addition, revealing that PLC contributes to the cytosolic calcium increase. PLC is known to stimulate IP_3_R in the ER membrane through the synthesis of IP_3_. Thus, to determine whether this pathway is important for H_2_O_2_-induced calcium increase, cells were pre-incubated with 50 *μ*M 2-APB (an IP_3_R inhibitor), which also led to significantly reduced H_2_O_2_-induced cytosolic calcium (Figures 2e and f). In addition, we assessed the role of SOC channels in this calcium influx by knocking down STIM1 with shRNA (Figures 2i and j). si-STIM1 inhibited the elevation of the right peak but not the left peak of calcium influx, indicating that SOC channels affect extracellular calcium influx without altering ER Ca^2+^ store release (Figures 2g and h). Together, these results support the notion that PLC induces a signalling cascade through IP_3_ and the subsequent stimulation of SOC channels to mediate H_2_O_2_-induced cytosol calcium increase.

**Figure 3:**
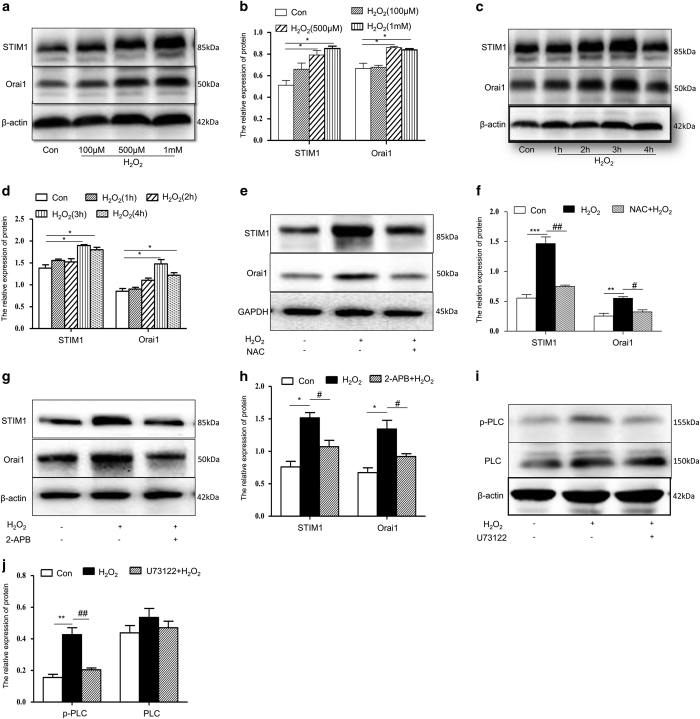
SOC participates in the H_2_O_2_-induced disturbance of calcium homoeostasis. (**a**–**h**) Western blot for STIM1 and Orai1 protein. Cells were stimulated with H_2_O_2_ at varying concentrations (100 *μ*M, 500 *μ*M or 1 mM) for 4 h (**a** and **b**) or were stimulated for varying periods of time (1, 2, 3 or 4 h) with 500 *μ*M H_2_O_2_ (**c** and **d**). Cells were pre-treated with 100 *μ*g/ml NAC (**e** and **f**) or 50 *μ*M 2-APB (**g** and **h**) or 10 *μ*M U73122 (**i** and **j**) for 21 h, then co-incubated with 500 *μ*M H_2_O_2_ for 3 h. PLC*γ*1 and p-PLC*γ*1 were detected by western blot. Shown are representative results from one of three independent experiments. *P<0.05, **P<0.01, ***P<0.001, compared with the control; ^#^*P*<0.05, ^##^*P*<0.01, compared with the H_2_O_2_-treated group.

To further validate proteomic changes that might be responsible for the H_2_O_2_-induced cytosolic calcium increase, the levels of some related proteins were assessed via western blot. Cells were exposed to H_2_O_2_ at concentrations of 100, 500 or 1000 *μ*M for 4 h or 500 *μ*M for 1, 2, 3 or 4 h. The results revealed that both STIM1 and Orai1 protein levels increased in a H_2_O_2_ concentration- and time-dependent manner (Figures 3a–d). Furthermore, NAC and 2-APB reversed this H_2_O_2_-induced increase in STIM1 and Orai1 levels (Figures 3e–h).

Because the PLC–IP_3_R pathway triggers SOC channels, we tested the levels of phosphorylated and total PLC*γ*1 after H_2_O_2_ treatment. Our results showed that H_2_O_2_ did not stimulate an increase in total PLC*γ*1 protein, but the levels of phosphorylated protein increased. These changes were significantly attenuated by the PLC*γ*1 inhibitor U73122 (Figures 3i and j).

### HMGB1 secretion and translocation are Ca^2+^ dependent

**Figure 4:**
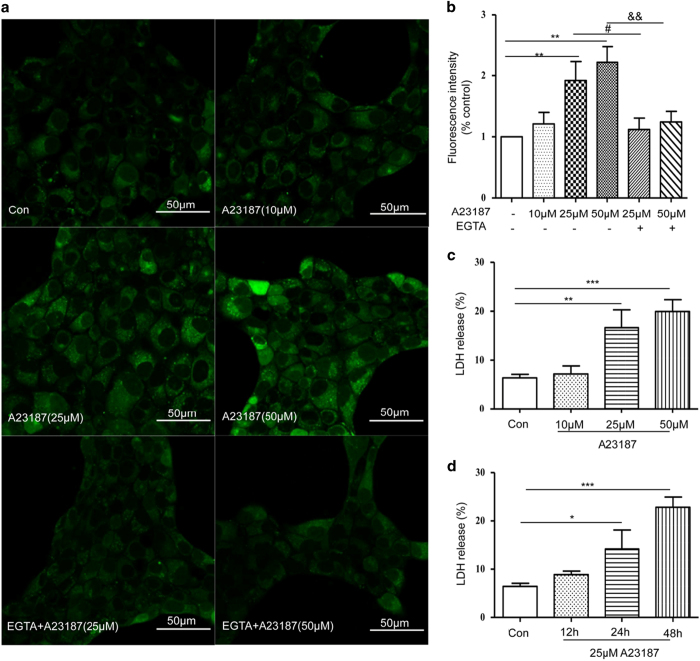
A23187-induced cell injury. (**a** and **b**) Intracellular Ca^2+^ levels at 24 h after A23187 treatment (10, 25, 50 *μ*M) or in the presence of 1 mM EGTA plus A23187 treatment (25 *μ*M, 50 *μ*M) visualized by confocal microscopy. (**c** and **d**) The rate of cell injury assessed using the Cytotoxicity LDH Assay Kit. Shown are representative results from one of three independent experiments. *P<0.05, **P<0.01, ***P<0.001, compared with the control; ^#^*P*<0.05, compared with the 25 *μ*M A23187-treated group; ^&&^*P*<0.01, compared with the 50 *μ*M A23187-treated group.

To determine whether intracellular calcium overload could induce cell injury, cells were treated with different concentrations (10, 25 and 50 *μ*M) of A23187, a calcium ionophore and intracellular Ca^2+^ levels were examined using Fluo-4/AM. Intracellular fluorescence signals gradually increased from 10 to 50 *μ*M in a concentration-dependent manner with A23187 treatment (Figures 4a and b). However, the bright fluorescence signals induced by A23187 were dampened by co-treatment with 1 mM EGTA, a calcium chelator. We also measured the release of LDH into the media under these conditions by ELISA assay (Figures 4c and d) and observed a significant concentration- and time-dependent increase with the A23187 treatment (Figures 4c and d). These results indicate that intracellular calcium overload induces cellular injury.

**Figure 5:**
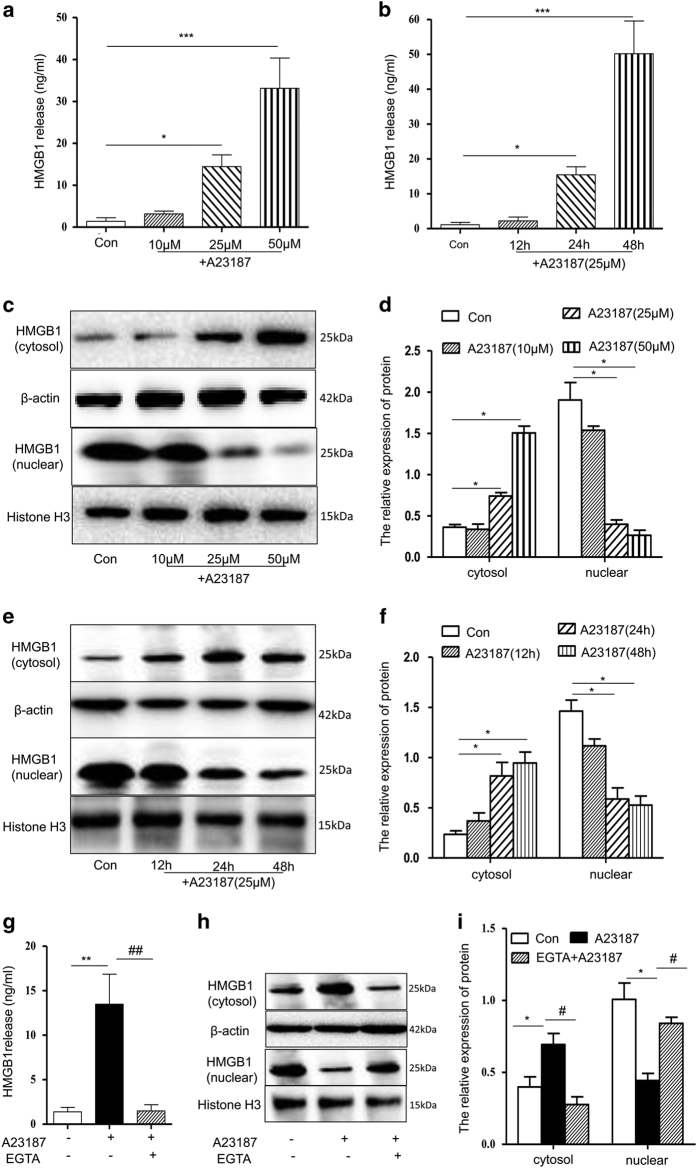
A23187-induced HMGB1 translocation and release in hepatocytes. (**a** and **b**) HMGB1 contents in culture media were determined using ELISA. (**c**–**f**) HMGB1 protein levels in cytosolic/nuclear fractions were measured by western blot. After incubation with A23187 (10, 25 or 50 *μ*M) for 30 min followed by 24 h without A23187 (**a**, **c** and **d**) or 25 *μ*M A23187 for 30 min followed by 12, 24 or 48 h without A23187 (**b**, **e** and **f**), the media and cells were harvested. (**g**) Cells were incubated with both 25 *μ*M A23187 and 1 mM EGTA for 30 min, and HMGB1 levels were measured in culture media by ELISA. (**h** and **i**) HMGB1 levels in cytosolic/nuclear fractions were measured by western blot. Shown are representative results from one of three independent experiments. *P<0.05, **P<0.01, ***P<0.001, compared with the control; ^#^*P*<0.05, ^##^*P*<0.01, compared with the A23187-treated group.

Similarly, HMGB1 levels in the cellular media increased with increasing concentrations of A23187 treatment (10, 25 or 50 *μ*M) in a concentration-dependent manner and also markedly increased at 24 and 48 h in a time-dependent manner (Figures 5a and b). Under the same conditions, the HMGB1 levels in the nucleus gradually decreased after A23187 treatment (10, 25 or 50 *μ*M), consistent with a corresponding increase in cytosolic levels (Figures 5c and d). Similar results were observed with different time periods of A23187 incubation (Figures 5e and f). Furthermore, in cells treated with 25 *μ*M A23187 combined with 1 mM EGTA, HMGB1 levels in the media and cytosol significantly decreased, coincident with an increase in nuclear levels (Figures 5g–i). Together, these results indicate that A23187 induces HMGB1 translocation and release through an increase in intracellular calcium.

**Figure 6:**
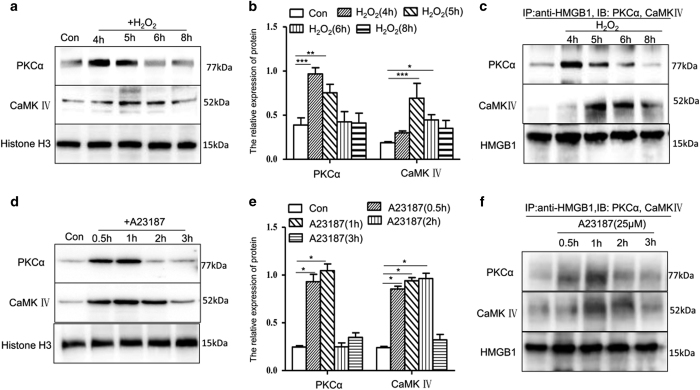
Intracellular calcium mediates the translocation and release of HMGB1 by PKC*α* and CaMKIV. (**a**)The nuclear levels of PKC*α* and CaMKIV were examined by western blot after H_2_O_2_ treatment (**a **and **b**) or A23187 treatment (**d** and **e**). (**c** and **f**) The cells were stimulated with H_2_O_2_ or A23187, and nuclear extracts were harvested at each time point, immunoprecipitated with anti-HMGB1 and protein A agarose, and subjected to western blot analysis for PKC*α* and CaMKIV expression. Shown are representative results from one of three independent experiments. *P<0.05, **P<0.01, ***P<0.001, compared with the control.

It is reported that PKC*α* and CaMKIV are involved in HMGB1 phosphorylation and release.^26,34^ To elucidate whether HMGB1 release from hepatocytes under oxidative stress is also dependent on these kinases, we examined changes in nuclear PKC*α* and CaMKIV levels and their interaction with HMGB1 after H_2_O_2_ exposure. The content of PKC*α* in the nucleus after H_2_O_2_ treatment increased at approximately 4–5 h, then decreased from 6 h. CaMKIV content increased at approximately 5–6 h and went down at 8 h (Figures 6a and b). The results of immunoprecipitation analysis of HMGB1 with PKC*α* or CaMKIV showed that both PKC*α* and CaMKIV can directly interact with HMGB1 in the nucleus over different time periods (Figure 6c).

Owing to the calcium-dependence of PKC*α* and CaMKIV activity, we also measured the role of A23187 in the interactions of PKC*α* and CaMKIV with HMGB1. The results showed that the nuclear levels of PKC*α* increased at 0.5 and 1 h but decreased at 2–3 h after A23187 treatment. Additionally, the levels of CaMKIV increased at 0.5–2 h and went down at 3 h after A23187 treatment. This indicates that both kinases are induced by A23187 treatment but that CaMKIV induction persists longer than PKC*α* induction (Figures 6d and e). To observe whether the interactions of PKC*α* or CaMKIV with HMGB1 in the nucleus are also regulated by calcium, nuclear extracts isolated from cells treated with or without A23187 were immunoprecipitated with anti-HMGB1. Both PKC*α* and CaMKIV could be observed in the resulting western blot (Figure 6f). These results suggest that PKC*α* and CaMKIV directly bind to HMGB1 in A23187-treated cells.

### Effects of 58-F on the PLC*γ*1–IP_3_R–SOC signalling pathway in the H_2_O_2_-induced [Ca^2+^]_i_ increase

**Figure 7:**
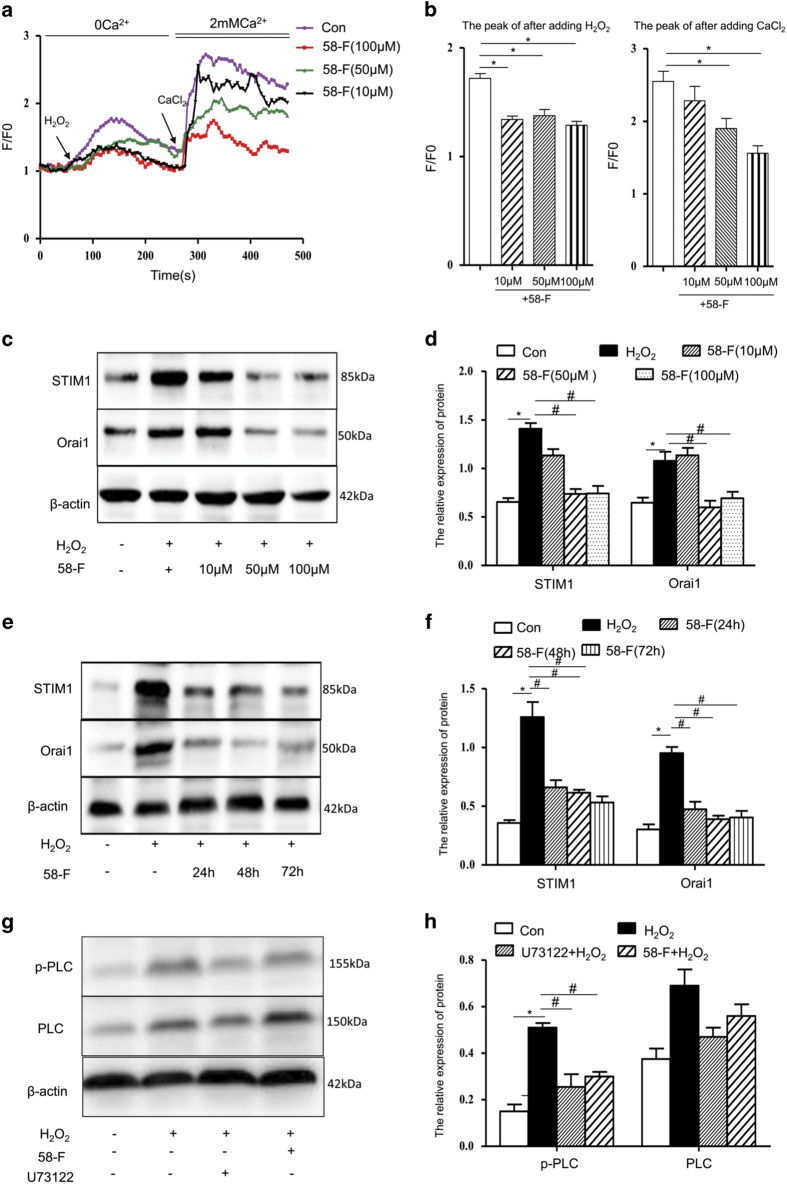
58-F reduces the H_2_O_2_-induced [Ca^2+^]_i_ increase. (**a**) Cells were pre-treated with 10, 50 or 100 *μ*M 58-F for 24 h, followed by stimulation with 500 *μ*M H_2_O_2_ in a Ca^2+^-free buffer and the subsequent addition of 2 mM CaCl_2_ into the media. (**b**) The quantification of intracellular Ca^2+^ was performed at two peaks after adding H_2_O_2_ and CaCl_2_. (**c**–**f**) Cells were pre-treated with 10, 50 and 100 *μ*M 58-F for 21 h, then co-incubated with 500 *μ*M H_2_O_2_ for 3 h (**c** and **d**) or with 50 *μ*M 58-F for 24, 48 and 72 h. (**e** and **f**) Western blot analysis of STIM1 and Orai1 proteins. (**g** and **h**) Cells were pre-treated with 10 *μ*M U73122 or 5 0 *μ*M 58-F for 21 h, then co-incubated with 500 *μ*M H_2_O_2_ for 3 h, followed by western blot analysis of p-PLC*γ*1 and PLC*γ*1 proteins. Shown are representative results from one of three independent experiments. *P<0.05, compared with the control; ^#^*P*<0.05, compared with the H_2_O_2_ treatment group.

Recently, we reported that 58-F protects against ROS-induced liver injury. In addition, we also showed that H_2_O_2_ could induce apoptosis by disrupting cellular calcium homoeostasis.^33,35^ The [Ca^2+^]_i_ increase by H_2_O_2_ prompted us to investigate the possibility that the inhibition of Ca^2+^ influx by 58-F is at least partly due to the suppression of the PLC*γ*1–IP_3_R–SOC signalling pathway. The level of calcium entry into cells with or without 58-F pretreatment was detected by calcium imaging and confocal microscopy. In agreement with Figure 2, the stimulation of hepatocytes with 500 *μ*M H_2_O_2_ in a calcium-free buffer led to an increase in cytosolic calcium due to the release of calcium from ER stores. After replacing the extracellular medium with a 2 mM CaCl_2_ solution, a further increase in cytosolic calcium through the PM was apparent, due to the depletion of ER stores. Notably, the fluorescence intensity after adding H_2_O_2_ and CaCl_2_ was attenuated by pretreatment with 50 or 100 *μ*M 58-F (Figures 7a and b).

To assess the effects of 58-F on the protein levels of members of the PLC*γ*1–IP_3_R–SOC signalling pathway, we measured the levels of STIM1, Orai1, PLC*γ*1 and p-PLC*γ*1 following treatment. The H_2_O_2_-induced increased levels of both STIM1 and Orai1 were reduced by pretreatment with 58-F at concentrations ranging from 10 to 100 *μ*M or by pretreatment with 50 *μ*M 58-F for different periods of time (24, 48 or 72 h) (Figures 7c–f). Furthermore, similar to the effects observed with U73122, 58-F significantly attenuated the increased levels of p-PLC*γ*1 induced by H_2_O_2_ without affecting the levels of total PLC*γ*1 (Figures 7g and h). These findings suggest that 58-F suppresses the H_2_O_2_-induced [Ca^2+^]_i_ increase through PLC*γ*1-mediated SOC channels.

### Effects of 58-F on the translocation and release of HMGB1 induced by H_2_O_2_

**Figure 8:**
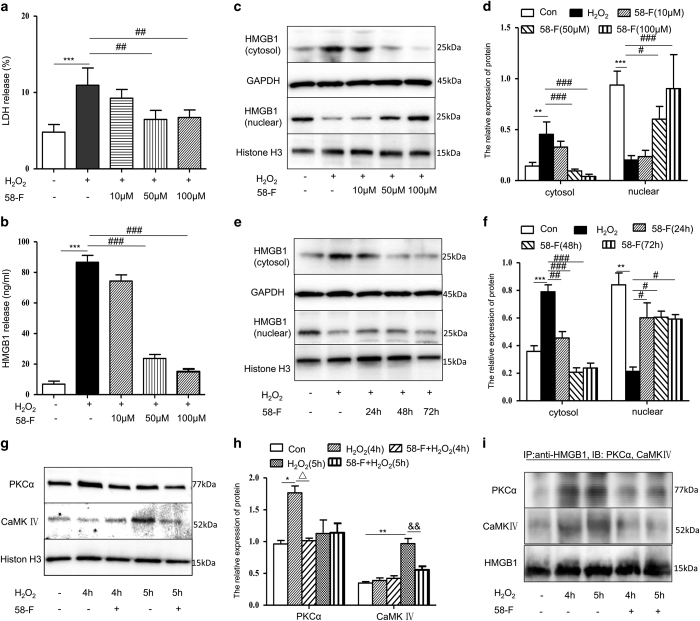
58-F reduces the H_2_O_2_-induced translocation and release of HMGB1. (**a**) LDH release of cells after treatment with 10, 50 or 100 *μ*M 58-F for 24 h. (**b**) HMGB1 levels in culture media were measured by ELISA. Cells were pre-treated with 50 *μ*M 58-F for 16 h, then co-incubated with 500 *μ*M H_2_O_2_ for 8 h. (**c** and **d**) HMGB1 protein levels in the cytosolic/nuclear fractions were measured by western blot. Cells were pre-treated with 10, 50 or 100 *μ*M 58-F for 16 h, then co-incubated with H_2_O_2_ for 8 h. (**e** and **f**) HMGB1 protein levels in the cytosolic/nuclear fractions were measured by western blot. Cells were pre-treated with 50 *μ*M 58-F for 24, 48 or 72 h. (**g** and **h**) PKC*α* and CaMKIV expression levels in the nucleus were measured by western blot. Cells were pre-treated with 50 *μ*M 58-F for 20 or 19 h, then continuously co-treated with 500 *μ*M H_2_O_2_ for 4 or 5 h. (**i**) Immunoprecipitation was performed with HMGB1 antibody, followed by western blot for PKC*α* or CaMKIV. Cells were pre-treated with 50 *μ*M 58-F for 20 or 19 h, then co-incubated with 500 *μ*M H_2_O_2_ for 4 or 5 h. Nuclear extracts were harvested, immunoprecipitated with anti-HMGB1 and protein A agarose, then subjected to western blot analysis for PKC*α* and CaMKIV. Shown are representative results from one of three independent experiments. *P<0.05, **P<0.01, ***P<0.001, compared with the control; ^#^*P*<0.05, ^##^*P*<0.01, ^###^*P*<0.001, compared with the H_2_O_2_-treated group; ^△^*P*<0.05, compared with the H_2_O_2_-treated 4 h group; ^&&^*P*<0.01, compared with the H_2_O_2_-treated 5 h group.

To confirm the cellular protective effect of 58-F, we examined the release of LDH from cells to media and the levels of HMGB1 in the media, cytosol and nucleus. The extracellular release of both LDH and HMGB1 was decreased after pretreatment with 10, 50 or 100 *μ*M 58-F (Figures 8a and b). Additionally, the translocation of HMGB1 from the nucleus to the cytosol was also suppressed in time- and dose-dependent manners (Figures 8c–f).

Likewise, the nuclear levels of PKC*α* and CaMKIV in cells pre-treated with 50 *μ*M 58-F were significantly less than those in cells with a single H_2_O_2_ treatment from 4 to 5 h (Figures 8g and h). To further assess the direct interaction of PKC*α* or CaMKIV with HMGB1, nuclear lysates were immunoprecipitated with anti-HMGB1, then subjected to western blot with PKC*α* or CaMKIV antibodies. The direct interaction of PKC*α* and CaMKIV with HMGB1 in the nucleus was inhibited by 58-F pretreatment (Figure 8i).

## Discussion

### A PLC*γ*1–IP_3_R–SOC signalling pathway is involved in the disturbance of calcium homoeostasis in hepatocytes induced by H_2_O_2_

Calcium signals, which can be induced by a variety of stimuli, control a myriad of functions in cells. Hepatocytes can increase their cytoplasmic Ca^2+^ concentration in two ways: release from intracellular storage pools, primarily ER,^36^ and the entry of extracellular calcium to maintain adequate Ca^2+^ stores. It is well established that PLC*γ*1 undergoes phosphorylation on tyrosine residues in response to H_2_O_2_ treatment.^20,21^ Under either physiological or pathological conditions, the activation of the PLC*γ*1 pathway produces IP_3_, which binds to its receptor IP_3_R in the ER and mobilizes Ca^2+^ out of ER. Subsequently, SOC channels are activated, leading to Ca^2+^ influx and the replenishing of ER stores. In our study, the role of PLC*γ*1-IP_3_R signalling in SOC was examined, and the results showed that H_2_O_2_-induced elevated [Ca^2+^]_i_ was almost abolished and that the increased phosphorylation of PLC*γ*1 was reduced when cells were pre-treated with the generic PLC inhibitor U73122 or the IP_3_R inhibitor 2-APB. These findings are in agreement with earlier publications reporting that the phosphorylation and activation of PLC by a sulfhydryl oxidation-dependent mechanism, which leads to increased IP_3_ synthesis and subsequent activation of the IP_3_ receptor, induces the release of Ca^2+^ from intracellular stores^37,38^ and that the H_2_O_2_-induced [Ca^2+^]_i_ rise could be prevented by U73122 or 2-APB.^39^ SOC is defined as enhanced Ca^2+^ import from the extracellular space after depletion of calcium in the ER.^6,40^ Among all Ca^2+^-permeable channels confirmed to be expressed in hepatocytes, SOC channels are the principal pathway for Ca^2+^ influx through the PM.^35,41^ To confirm whether SOC participates in H_2_O_2_-induced Ca^2+^ influx, we carried out a series of experiments. First, our data demonstrate that H_2_O_2_ elicits an increase in intracellular [Ca^2+^]_i_ in the absence of extracellular calcium, indicating that H_2_O_2_ can mobilize calcium out of the ER. After adding CaCl_2_, an apparent extracellular Ca^2+^ influx was observed, and the overall rise in calcium concentration significantly increased. Furthermore, the antioxidant NAC inhibited Ca^2+^ influx from the extracellular space. These findings are in agreement with the view that H_2_O_2_ triggers Ca^2+^ release into the cytoplasm in two steps: Ca^2+^ release from internal stores, followed by SOC from the extracellular supply. Second, the protein levels of STIM1 and Orai1 increased after H_2_O_2_ treatment, while si-STIM1 almost abolished the H_2_O_2-_induced Ca^2+^ influx without affecting the release of Ca^2+^ from the ER (Figure 2). Moreover, NAC was found to inhibit the H_2_O_2-_induced increase of STIM1 and Orai1 protein levels. These findings confirm that Ca^2+^ enters cells through activated SOC channels with H_2_O_2_ stimulation. Together, we conclude that calcium overload in hepatocytes caused by H_2_O_2_ occurs through the PLC*γ*1–IP_3_R–SOC signalling pathway.

### The increase of intracellular calcium can activate PKC*α* and CaMKIV to promote HMGB1 release

Extracellular HMGB1 is derived from either active secretion by innate immune cells or by passive release from dead or stressed cells as a late inflammatory mediator for infectious or noninfectious inflammation.^24^ The passive release of HMGB1 from dead/stressed cells is due to its export from the nucleus to the cytoplasm and subsequent release into the extracellular space due to increased cell membrane permeability.^23,42^ Youn and co-workers^43^ and Tsung Allan *et al.*^28^ have reported that HMGB1 release can be induced by A23187 in murine hepatocytes or in RAW264.7 cells and that this release is reduced by BAPTA, a chelator of Ca^2+^, indicating that Ca^2+^ plays a critical role in oxidative stress-induced HMGB1 release. Our results show that both HMGB1 and LDH contents in the media increased after H_2_O_2_ treatment and that HMGB1 levels in the media induced by H_2_O_2_ exposure are markedly reduced by pretreatment with either the antioxidant NAC or the calcium pathway inhibitors 2-APB or U73122. This confirms the finding that HMGB1 release from stressed/dead cells due to oxidative stress is regulated by calcium homoeostasis. To further verify the possibility that Ca^2+^ overload in the cytosol due to oxidative stress leads to HMGB1 release, we employed the calcium ionophore A23187. Our data show that increasing intracellular levels of calcium with A23187 results in the translocation of HMGB1 from the nucleus to the cytosol and its release to the extracellular space, which was markedly reduced by treatment with the calcium chelator EGTA.

Phosphorylation of serine residues within the HMGB1 nuclear localization signal may also contribute to the regulation of HMGB1 cytoplasmic translocation, which is a key step for its release into the extracellular space.^22,25^ Two calcium-dependent kinases, CaMKIV and PKC*α*, have been implicated in the regulation of HMGB1 phosphorylation and release.^27,43^ Signalling events upstream of PKC*α* and CaMKIV include Ca^2+^ release from the ER. We noted an increase in the levels of both PKC*α* and CaMKIV within the nucleus and an enhancement of their direct interaction with HMGB1 after A23187 stimulation. This is consistent with the results of H_2_O_2_ treatment, supporting the view that the H_2_O_2_-mediated increase in cytoplasmic Ca^2+^ is sufficient to activate CaMKIV and PKC*α* nuclear translocation and HMGB1 phosphorylation.

### 58-F intervention regulates intracellular calcium and reduces the release of HMGB1 induced by H_2_O_2_

Accumulating evidence directly implicates HMGB1 in various diseases, and it has been considered as a therapeutic target for sterile inflammation and infection.^44^ We recently reported that 58-F protected against ROS-induced liver injury, but the mechanism was still to be elicited. The results reported in this work indicate that 58-F inhibits calcium entry through SOC channels triggered by the PLC*γ*1-IP_3_R signalling pathway in response to oxidative damage. We also showed that 58-F suppresses HMGB1 translocation from the nucleus to the cytoplasm and its eventual extracellular release by inhibiting the activation of PKC*α* and CaMKIV.

## Conclusion

**Figure 9:**
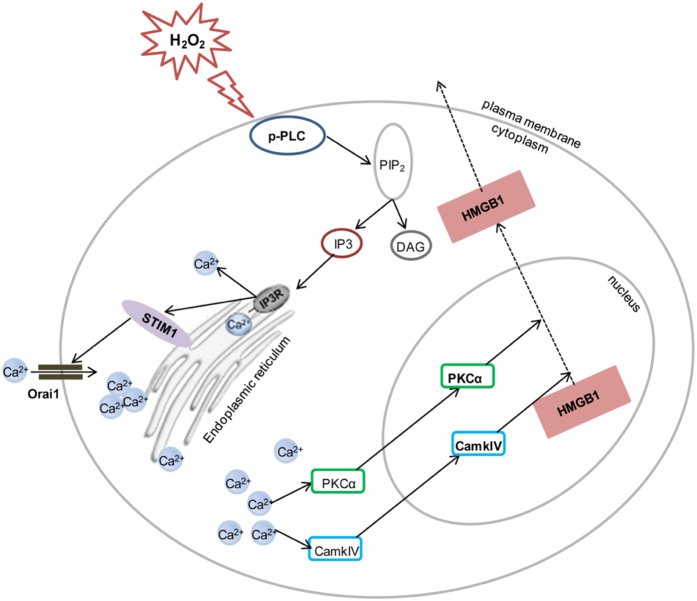
HMGB1 release regulated by the PLC*γ*1–IP_3_R–SOC signalling pathway during calcium overload in hepatocytes. The proposed model showing the release of HMGB1 following H_2_O_2_ treatment. H_2_O_2_ activates the PLC*γ*1–IP_3_R–SOC signalling pathway, leading to calcium influx and the activation of calcium-dependent kinases PKC*α* and CAMKIV and triggering HMGB1 phosphorylation. Phosphorylated HMGB1 translocates from the nucleus to the cytoplasm and is ultimately released into the extracellular space.

In summary (Figure 9), we determined the role of the PLC*γ*1–IP_3_R–SOC signalling pathway in the regulation of calcium influx in cells undergoing oxidative stress and identified calcium homoeostasis in hepatocytes as a key mechanism regulating HMGB1 translocation from the nucleus to the cytoplasm for extracellular release. Our data further support the idea that calcium-dependent kinases PKC*α* and CaMKIV participate in HMGB1 phosphorylation, a key step leading to HMGB1 release. On the basis of these findings, 58-F, a flavanone extracted from *O. japonicus*, was shown to interfere with calcium overload caused by the response of the PLC*γ*1–IP_3_R–SOC signalling pathway to oxidative stress and with the PKC*α*- and CaMKIV-mediated regulation of HMGB1 release. These findings may be important for designing therapies to prevent hepatocytes from oxidative stress-induced injury/death.

## Materials and methods

### Drugs and reagents

Thirty percent H_2_O_2_ was purchased from Sinopharm Chemical Reagent Co. (Shanghai, China). 58-F was purchased from Shanghai Yilin Biotechnology Co., Ltd (Shanghai, China). A23187 was purchased from Abcam (Cambridge, MA, USA). 2-Aminoethoxydiphenyl borate(2-APB) and NAC were purchased from Sigma (St. Louis, MO, USA). EGTA was purchased from Amresco (Solon, OH, USA). U73122 was purchased from Selleck (Houston, TX, USA). Dulbecco’s modified Eagle’s medium (DMEM), fetal calf serum (FBS) and penicillin–streptomycin were purchased from Gibco (Carlsbad, CA, USA). Hanks and D-Hanks were purchased from Gino Biological Pharmaceutical Co., Ltd (Hangzhou, China). Fluo-4/AM, Pluronic F-127 and Cytotoxicity LDH Assay Kit were purchased from Dojindo (Kumamoto, Japan). Rabbit anti-rat antibodies against STIM1, PLC*γ*1, p-PLC*γ*1, GAPDH, β-actin were purchased from Santa Cruz Biotechnology (Dallas, TX, USA); Orai1, HMGB1, PKC*α*, CaMKIV, Histone H3 were purchased from Abcam. Goat anti-rabbit horseradish peroxidase-conjugated secondary antibody was purchased from Shanghai Excell Biotechnology Co., Ltd (Shanghai, China). The BCA protein assay kit, SDS-PAGE and Protein A agarose were purchased from (Shanghai, China). Enhanced chemiluminescence detection system was purchased from Millipore (Billerica, MA, USA). The HMGB1 enzyme-linked immunosorbent assay (ELISA) kit was purchased from Chondrex (Redmond, WA, USA). Nuclear and cytoplasmic extraction reagents was purchased from Shanghai Yisheng Biotechnology Co., Ltd (Shanghai, China).

### Cellular culture and treatment

BNL.Cl_2_ cells were cultured in DMEM medium supplemented with 10% FBS, 1% penicillin and streptomycin at 37°C with 5% CO_2_, and the culture media were changed once every 2 days. At 80–90% confluence, cells were treated with various agents in medium with 2% FBS. For H_2_O_2_ treatment, cells were incubated with 100 *μ*M, 500 *μ*M or 1 mM H_2_O_2_ for 1, 2, 3 or 4 h. For NAC treatment, cells were pre-treated with 100 *μ*g/ml NAC for 24 h. For inhibition experiments, the cells were pre-treated with 50 *μ*M 2-APB or 10 *μ*M U73122 for 24 h and then co-treated with 500 *μ*M H_2_O_2_ for 3 h. For RNAi experiments, cells were transfected with NC-siRNA or STIM1-siRNA. For A23187 treatment, cells were treated with 10, 25 or 50 *μ*M A23187 for 30 min; then, the medium was replaced with new DMEM with 2% FBS for 12, 24 or 48 h. Cells were co-treated with 25 *μ*M A23187 and EGTA (25 *μ*M) for 30 min. For 58-F administration, the cells were pre-treated with 58-F at 10, 50 or 100 *μ*M for 24, 48 or 72 h and then exposed to 500 *μ*M H_2_O_2_ for 3 h.

### Measurement of intracellular Ca^2+^ concentration

Intracellular Ca^2+^ was monitored using the Ca^2+^-sensitive fluorescent indicator-Fluo-4/AM by confocal laser scanning microscopy. The cells were cultured in confocal dishes (10^5^ cells/dish), then loaded with Fluo-4/AM (5 *μ*M) and F127 (0.4%) at 37 °C for 30 min in darkness with modified Hank’s Buffered Salt Solution (HBSS) containing 25 mM Hepes, 150 mM NaCl, 5 mM KCl, 2 mM CaCl_2_, 0.4 mM MgCl_2_ and 25 mM d-glucose (pH 7.4). They were rinsed twice in HBSS and kept at room temperature for 10 min to allow the de-esterification of Fluo-4/AM. Then, cells were maintained in D-Hanks (Ca^2+^-free) buffer for 10 min before Ca^2+^ imaging. The green fluorescence of Fluo-4/AM was excited by an argon laser at 488 nm, and the emitted fluorescence was recorded through a 525 nm channel. The image was recorded every 10 s. For imaging with Fluo-4/AM, [Ca^2+^]_i_ changes are presented as *F*/*F*_0_ ratios after background subtraction, where *F* was the change in fluorescence signal intensity, and *F*_0_ was the baseline before stimulus application.

### LDH cytotoxicity assay

LDH release, which could reflect cell membrane integrity, was detected with an assay kit (Dojindo) according to the manufacturer’s instructions. Briefly, cells were cultured in 96-well microplates (7×10^3^ cells/well) for 24 h in a CO_2_ incubator and then incubated with different treatments. First, 10 *μ*l of Lysis Buffer was added to the well to induce maximum LDH release. After 30 min in a CO_2_ incubator, 100 *μ*l of Working Solution was added into each well, and the plates were incubated in darkness at room temperature for 30 min. Subsequently, 50 *μ*l of Stop Solution was added to each well, and the absorbance was measured at 490 nm using a microplate reader. The calculation was as follows: LDH release (%)=[A-C/B-C]×100%, where A is the absorbance of treated samples, and B and C are the absorbance of the maximum and the minimum, respectively.

### ELISA assay

The cell culture medium was collected and used to measure HMGB1 levels by ELISA kits according to the manufacturer’s suggested protocol. Absorbance at 450 nm was measured using a microplate reader.

### Preparation of cell extracts

Nuclear and cytosolic extracts were prepared using nuclear and cytoplasmic extraction reagents, according to the manufacturer’s instructions. The total protein extract was lysed in RIPA buffer and phenylmethylsulfonyl fluoride. The protein concentrations were determined by bicinchoninic acid assay.

### Western blot analysis

Equal amounts of total cellular protein (20 *μ*g per sample) were separated by SDS-PAGE and transferred to PVDF membranes that were blocked with 5% non-fat milk for 2 h and incubated with the primary antibodies overnight at 4 °C. The primary antibodies (diluted at 1 : 1000) used were as follows: anti-STIM1, anti-Orai1, anti-PLC*γ*1, anti-p-PLC*γ*1, anti-HMGB1, anti-PKC*α*, anti-CaMKIV anti-GAPDH, anti-β-actin, and anti-Histone H3 (1 : 500). The membranes were then washed three times (10 min/wash) with TBST and incubated with a 1 : 3000 dilution of goat anti-rabbit horseradish peroxidase-conjugated secondary antibody for 1 h at room temperature. After the final wash, the immunoreactive bands were detected on Fluor Chem E (Protein Simple) by enhanced chemiluminescence. The densities of bands were analysed using ImageJ software and expressed as ratios to *β*-actin or GAPDH or Histone H3.

### Immunoprecipitation

A total of 500 *μ*g of nuclear extract was incubated with 2 *μ*g of anti-HMGB1 at 4 °C overnight on a rotator. Next, 30 *μ*l of Protein A Agarose was spun briefly in a microcentrifuge at 500 *g* for 30 s and washed three times in PBS, then resuspended in 200 *μ*l of PBS. A 40 *μ*l slurry of Protein A agarose was added to each sample, followed by incubation for an additional 3 h at 4 °C on a rotator. The samples were spun briefly in a microcentrifuge at 500 *g* for 2 min and washed three times in PBS. Finally, the samples were resuspended in 40 *μ*l of loading buffer for future analysis.

### RNAi

We utilized the Smartpool siRNA from Dharmacon (Lafayette, CO, USA) that consists of four separate siRNA sequences against STIM1: GCGACTTCTGAAGAGTCTACC, GCTGCTGGTTTGCCTATATCC, GCGGTTTCCAGATTGTCAATA and GGATTTGACCCATTCCGATTC, and a control siRNA NC: TTCTCCGAACGTGTCACGT. The LV3(H1/RFG&Puro)-STIM1 and NC constructs were constructed and identified by Shanghai UsenLab Biotechnology Co., Ltd. (Shanghai, China). Cells were plated in a six-well plate or confocal dish in 10% DMEM without antibiotics, resulting in 80% confluence before transfection. In separate tubes, 1.5 *μ*g of each plasmid was diluted in 250 *μ*l serum-free DMEM. Each solution was combined with 5 *μ*l of Lipofectamine 2000 and mixed gently, and the final transfection mixture was incubated for 20 min at room temperature. The cells were transfected with STIM1-siRNA or NC-siRNA according to the manufacturer’s protocol (Invitrogen, Carlsbad, CA, USA). Transfection efficiency was determined at 24 h by fluorescence microscopy, and mRNA and protein expression levels were measured 48 h after transfection.

### Statistical analysis

Experiments were carried out in triplicate, and statistical analysis was performed using SPSS software. Significance between two groups was assessed using the paired or non-paired Student’s *t*-test (*t*-test), and significance among multiple groups was assessed using a one-factor analysis of variance (ANOVA) with the Dunnett’s *post hoc* test. *P*<0.05 was considered as a statistically significant difference.
